# Optimal proprioceptive training combined with rehabilitation regimen for lower limb dysfunction in stroke patients: a systematic review and network meta-analysis

**DOI:** 10.3389/fneur.2024.1503585

**Published:** 2024-12-20

**Authors:** Kaiqi Zheng, Li Li, Yahui Zhou, Xiaokun Gong, Gangbin Zheng, Liang Guo

**Affiliations:** ^1^School of Physical Education and Sports Science, South China Normal University, Guangzhou, Guangdong, China; ^2^School of Health and Kinesiology, Georgia Southern University, Statesboro, CA, United States; ^3^Physical Education Department, Guangdong Pharmaceutical University, Guangzhou, Guangdong, China

**Keywords:** stroke, proprioceptive training, lower limb function, network meta-analysis, closed kinematic chain exercise

## Abstract

**Background:**

This study aims to evaluate the optimal rehabilitation regimen for lower limb dysfunction in stroke patients by analyzing the effects of proprioceptive training (PT) in combination with different rehabilitation interventions.

**Methods:**

Randomized controlled trials (RCTs) published up to April 23, 2024, were searched from PubMed, Embase, Cochrane Library, Web of Science, CNKI, Wanfang, VIP, and SinoMed. The quality of the included studies was assessed using the Cochrane Risk of Bias tool (ROB 2.0). Network meta-analysis was performed via R studio and STATA 15.0.

**Results:**

A total of 64 RCTs involving 4,084 stroke patients with lower limb dysfunction were included. For balance ability in stroke patients, PT in combination with motor relearning programme (PT + MRP) demonstrated the optimal rehabilitation effect [surface under the cumulative ranking curve (SUCRA) 77.94%]. For lower limb motor function, PT in combination with closed kinematic chain exercises (PT + CKCE) was most effective (SUCRA 88.39%). For walking ability, PT in combination with visual feedback training (PT + VFT) was superior (SUCRA 96.61%). Cluster analysis indicated that PT + CKCE and PT + RT1 were the optimal rehabilitation regimens for lower limb dysfunction in stroke patients.

**Conclusion:**

PT+MRP was the optimal rehabilitation regimen for improving balance ability in stroke patients; PT+CKCE was the best for enhancing lower limb motor function; and PT+VFT was most effective for improving walking ability. Overall, PT+CKCE and PT+RT1 represented the optimal rehabilitation regimens for lower limb dysfunction in stroke patients, while PT+RT1 is most effective within 5 days of stroke onset.

**Systematic review registration:**

https://www.crd.york.ac.uk/PROSPERO/#recordDetailsCRD42024548889, PROSPERO CRD42024548889.

## 1 Introduction

Among adults worldwide, stroke ranks third in terms of disability and is the second most common cause of death (1). The high incidence, disability rate, recurrence rate, and mortality associated with stroke have increasingly become a concerning global social issue. Among stroke survivors, lower limb hemiplegia is a common post-stroke sequela, which is clinically manifested by stiffness, contracture, and pain in the affected limb (2). This sequela often leads to decreased muscle strength, restricted joint mobility, balance disorders, abnormal gait, and other lower limb dysfunctions in patients during daily work and life (3), causing significant physical and psychological distress. Given the substantial harm that stroke causes to society and individuals, the rehabilitation of lower limb dysfunction following stroke is crucial.

Current research indicates that rehabilitation measures such as acupuncture, electrical stimulation, core stability training, and proprioceptive training (PT) are commonly used for lower limb rehabilitation in stroke patients in clinical practice (4–7) PT has been reported to have particularly outstanding therapeutic effects (8). Proprioception is the sense of the movement and position of the body and limbs in space (9). PT is an exercise method for improving the body's proprioceptive functions, such as muscle sensation, postural balance, and joint stability (8). It is a complex neuromuscular process involving the internal awareness of body posture and movement (10). Studies have shown that at least 30% of stroke patients experience proprioceptive deficits, which are negatively correlated with limb function, motor ability, and independence in daily life (11). This explains why PT achieves superior efficacy in treating post-stroke lower limb dysfunction compared to other rehabilitation interventions. Various clinical studies have also confirmed that PT can enhance lower limb proprioception in stroke patients and promote the recovery of lower limb function. For example, Chae et al. (7) found that stroke patients who received PT as a therapeutic measure exhibited better recovery in balance and walking function compared to those who underwent conventional rehabilitation. Similarly, a study by Mao et al. (12) demonstrated that PT was superior to conventional rehabilitation therapy in restoring lower limb motor function in stroke patients. Over the past two decades, using PT to treat lower limb dysfunction in stroke patients has matured and achieved favorable outcomes in clinical practice.

To further mitigate the impact of lower limb dysfunction in stroke patients, clinicians have combined PT with other commonly used rehabilitation interventions for stroke-induced lower limb dysfunction, aiming to achieve better therapeutic results. For instance, Shim et al. (13) found that combining PT with electrical stimulation therapy was more effective in the rehabilitation of balance and gait abilities in stroke patients than PT alone. Additionally, the study by Kim and Kim (14) showed that combining thermotherapy with PT significantly outperformed traditional rehabilitation therapies in improving balance and gait abilities in stroke patients. These combination therapies, involving PT and other rehabilitation interventions, are generally more efficacious in the rehabilitation of lower limb dysfunction caused by stroke compared to either PT or conventional rehabilitation alone. However, there are currently no studies comparing the efficacy of PT in combination with other rehabilitation interventions.

Unlike standard meta-analyses that compare two therapies directly, network meta-analysis (NMA) can integrate direct, indirect, and mixed comparisons of data, allowing for the ranking of several therapies according to their efficacy (15). This study employed NMA to compare the efficacy of PT, conventional rehabilitation therapy, and PT in combination with other rehabilitation interventions in the recovery of lower limb function after stroke, so as to identify the optimal rehabilitation regimen and provide guidance for clinical practice.

## 2 Methods

This NMA was conducted as per the guidelines outlined in the Preferred Reporting Items for Systematic Reviews and Meta-Analyses (PRISMA) 2020 statement and the procedures detailed in the Cochrane Handbook (16). The protocol for this systematic review has been registered on the PROSPERO under registration ID CRD42024548889.

### 2.1 Search strategy

Eight electronic databases were searched thoroughly, including CNKI, VIP, Wanfang, SinoMed, PubMed, Embase, Cochrane Library, and Web of Science. In addition to the conventional English databases including PubMed, Embase, Cochrane Library, and Web of Science, we included four Chinese databases: CNKI, VIP, Wanfang, and SinoMed, to enhance the comprehensiveness of our literature search. The time period for the search was set as of April 23, 2024. To meet the requirements of each database, various search strategies were used. The comprehensive search techniques are given in Supplementary Table S1. This search strategy has certain limitations, such as excluding literature in languages other than Chinese and English and not excluding older studies.

### 2.2 Inclusion and exclusion criteria

The inclusion criteria were as follows: (1) Participants: Stroke patients with lower limb dysfunction at any stage and time of onset, regardless of gender, race, or nationality. (2) Interventions: PT alone or PT in combination with other rehabilitation interventions. (3) Control group: PT alone or conventional rehabilitation therapy. (4) Outcome measures: The outcome measures included the Berg Balance Scale (BBS), Fugl-Meyer Assessment-lower extremity (FMA-LE), and Timed Up-and-Go Test (TUGT). The BBS scale is used to assess the balance ability of stroke patients, with higher scores indicating better balance; the FMA-LE scale is used to evaluate lower limb motor function in stroke patients, with higher scores indicating better motor function; the TUGT is used to measure the walking ability of stroke patients, with shorter test times indicating better walking ability. (5) Study design: Only peer-reviewed randomized controlled trials (RCTs) with available and detailed data were included.

The exclusion criteria were as follows: (1) Reviews, meta-analyses, conference papers, replies, letters, guidelines, case reports, and animal experiments. (2) Studies with inaccessible full text. (3) Other reasons, including articles with inaccessible original data, incomplete data, or erroneous data.

### 2.3 Literature screening and data extraction

Studies were imported into EndNote X9 for literature screening. Two independent reviewers removed duplicates, and reviewed titles and abstracts. Based on inclusion and exclusion criteria, irrelevant studies were excluded. Then, the full texts of the remaining studies were checked to eliminate ineligible studies. In cases of disagreement, the two reviewers discussed or consulted with a third researcher. The primary data extracted from the studies included basic information on the articles (author, year of publication, country), patient information (age, sample size, disease course), intervention (interventions in the experimental and control groups), and outcome measures.

### 2.4 Quality assessment

The quality of the included studies was assessed by two researchers using the Cochrane Risk of Bias tool (ROB 2.0). The studies were categorized into “low risk,” “moderate risk,” or “high risk.” Any discrepancies in the assessment results were resolved through discussion with a third researcher.

The researchers evaluated the risk of bias in the studies based on the following five aspects: (1) bias arising from the randomization process, (2) bias due to deviations from intended interventions, (3) bias due to missing outcome data, (4) bias in outcome measurement, and (5) bias in selective reporting of outcomes.

### 2.5 Data analysis

Bayesian NMA was conducted by using the software, R Studio. Data preprocessing was performed using the “gemtc” package in Stata 15, which also plotted the network of relationships among the interventions. Data from the final included RCTs were analyzed using the “gemtc” and “coda” packages in R Studio 4.2.1. Using 50,000 sampling iterations and 20,000 burn-in iterations, a consistency model was built. The data were considered consistent if there was a difference of < 5 between the deviance information criterion (DIC) obtained from the iteration results and the DIC of the inconsistency model, suggesting that there was no substantial difference between the NMA results and the direct comparisons. The efficacy of various interventions was ranked using the surface under the cumulative ranking curve (SUCRA). To investigate the optimal intervention, a two-dimensional hierarchical clustering analysis was used, and publication bias for each outcome measure was evaluated.

## 3 Results

### 3.1 Search results

**Figure 1:**
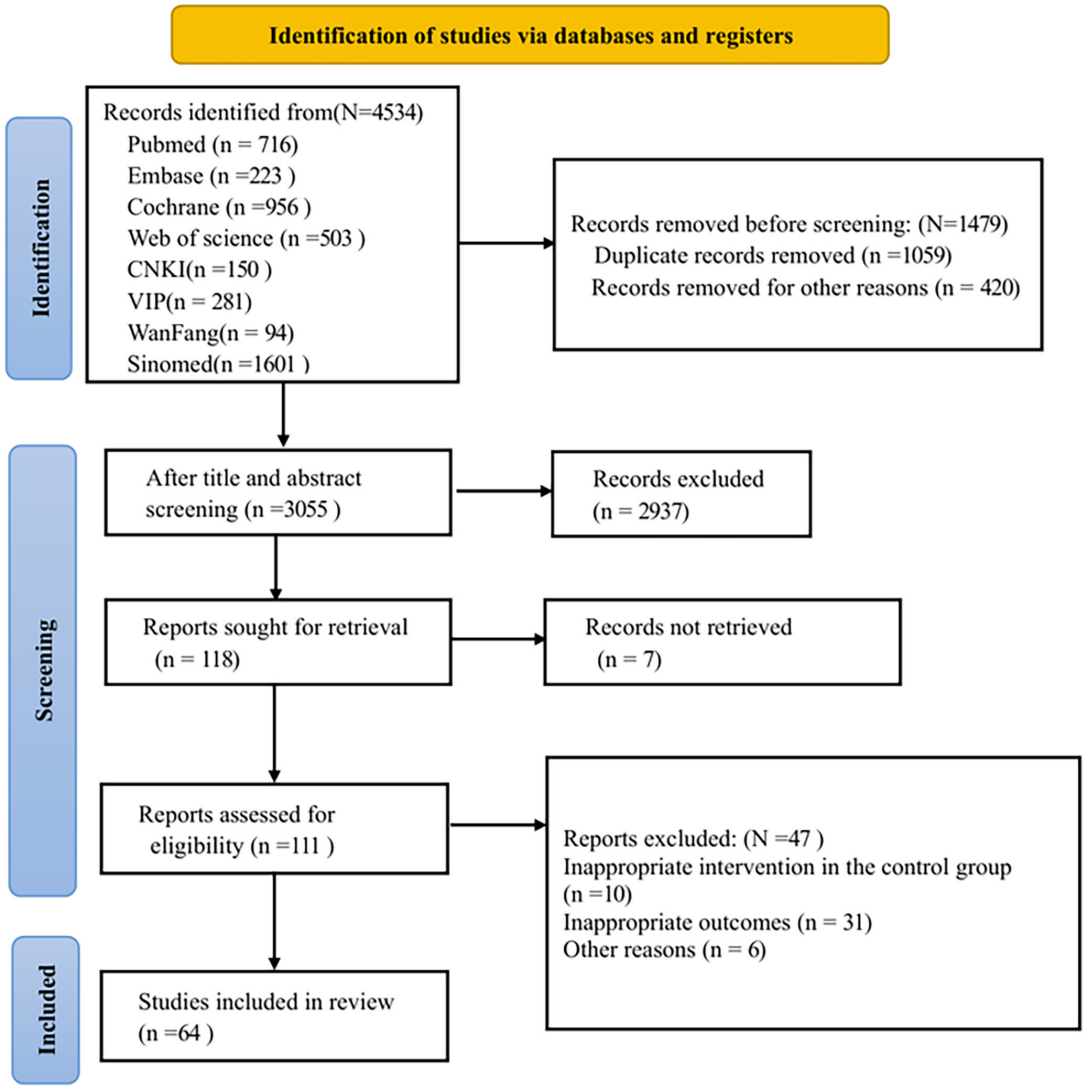
Flow chart of literature screening.

Initially, 4,534 records were searched based on the search strategy. After removing 1,059 duplicates, 420 records were excluded as reviews, animal experiments, non-RCTs, or conference papers, leaving 3,055 records. After screening titles and abstracts, 2,937 records were excluded. One hundred and eighteen articles were left for full-text review. Among these, the full texts of seven articles were not available; 10 articles had inappropriate control group interventions; 31 articles had inappropriate outcome measures, and six articles were excluded for other reasons. Ultimately, 64 RCTs (3, 7, 12–14, 17–75) were included in this study, all of which were published. Figure 1 shows the flow chart for the literature screening process in the study.

### 3.2 Characteristics of included studies

All 64 included studies were RCTs, involving a total of 4,084 stroke patients and 20 interventions. In the experimental groups, in addition to PT, there were 18 combined rehabilitation interventions. These 18 combined interventions were as follows: PT + dual-task training (PT + DTT), PT + closed kinematic chain exercises (PT + CKCE), PT + resistance training (PT + RT1), PT + core stability training (PT + CST), PT + acupuncture and moxibustion (PT + AM), PT + visual feedback training (PT + VFT), PT + electrical stimulation (PT + ES), PT + kinesio taping (PT + KT), PT + rope therapy (PT + RT2), PT + task-oriented training (PT + TOT), PT + robotic rehabilitation therapy (PT + RRT), PT + motor relearning programme (PT + MRP), PT + shock therapy (PT + ST), PT + static balance training (PT + SBT), PT + virtual reality training (PT + VRT), PT + thermal stimulation (PT + TS), PT + motor imagery training (PT + MIT), and PT + ankle-foot orthosis treatment (PT + AFOT). The basic characteristics of the included studies are detailed in Table 1.

**Table 1:** The basic characteristics of the included studies.

**ID**	**References**	**Year**	**Region**	**Sample size (TG-CG)**	**Age (TG)**	**Age (CG)**	**Duration (TG)**	**Duration (CG)**	**Intervention (TG)**	**Intervention (CG)**	**Outcomes**
1	Wang et al. (17)	2021	China	37–37	60.85 ± 6.32	61.46 ± 5.76	32.56 ± 4.93 d	33.48 ± 6.02 d	PT	C	①②
2	Wang et al. (18)	2023	China	36–36	70.26 ± 3.54	71.13 ± 3.61	7.3 ± 1.69 d	7.8 ± 1.82 d	PT + DTT	C	①②
3	Zhong et al. (19)	2021	China	31–31	56.01 ± 5.98	54.36 ± 6.08	①	①	PT + CKCE	C	①②
4	Jin et al. (20)	2018	China	30–30	50.33 ± 9.44	44.4 ± 7.2	4.10 ± 0.84 d	4.47 ± 0.97 d	PT + RT1	C	①②③
5	Zhang et al. (21)	2017	China	30–30	47.80 ± 11.46	46.62 ± 13.01			PT	C	①
6	Zhang (22)	2022	China	40–40	64.15 ± 2.26	63.98 ± 2.24			PT	C	①②
7	Zhang (23)	2022	China	35–35	61.12 ± 8.16	60.54 ± 8.23			PT	C	②③
8	Zhang (24)	2014	China	20–20	65.13 ± 5.38	62.36 ± 6.43			PT + CST	C	①②③
9	Yu et al. (25)	2018	China	51–50	62.5 ± 10.06	60.1 ± 11.09	14.00 ± 4.35 m	12.90 ± 3.31 m	PT	C	①③
10	Yang et al. (26)	2022	China	43–43	56.1 ± 7.4	55.8 ± 8.0	4.21 ± 1.02 m	4.03 ± 1.10 m	PT + AM	PT	①②③
11	Yang and Wu (27)	2016	China	60–60	59.5 ± 9.1	58.7 ± 10.4	3.6 ± 1.4 m	3.3 ± 2.1 m	PT	C	①②
12	Chen et al. (28)	2016	China	10–10	55.72 ± 17.36	51.34 ± 15.21	3.9 ± 1.3 m	4.2 ± 1.5 m	PT + ES	PT	②
13	Chen et al. (29)	2022	China	30–29	64 ± 8	64 ± 6	5.50 w	6.00 w	PT + AM	C	①
14	Cai et al. (30)	2016	China	20–20					PT	C	①
15	Xian et al. (31)	2023	China	30–30	54.22 ± 3.19	55.58 ± 3.54	22.51 ± 1.42 d	21.81 ± 1.22 d	PT	C	①②③
16	Su et al. (32)	2022	China	43–43	62.31 ± 5.24	62.42 ± 5.31	26.34 ± 6.38 d	26.25 ± 6.41 d	PT + MRP	PT	①
17	Zhu (33)	2020	China	40–40	60.4 ± 13.6	60.3 ± 13.4			PT + SBT	C	①
18	Wang et al. (34)	2024	China	40–40	68.23 ± 5.21	67.41 ± 5.62	11.85 ± 4.66 m	12.84 ± 4.69 m	PT + AM	C	②
19	Xiong and Hu (35)	2023	China	40–40	56.10 ± 4.20	54.70 ± 3.60	4.50 ± 1.90 m	4.30 ± 2.10 m	PT + ES	C	①②
20	Wang et al. (36)	2010	China	30–30	63.5 ± 10.5				PT	C	②
21	Wang et al. (37)	2023	China	40–40	60.05 ± 3.69	60.02 ± 3.72	56.87 ± 4.01 d	57.02 ± 3.99 d	PT + KT	PT	①②
22	Wang et al. (38)	2018	China	31–31	69.23 ± 10.85	69.94 ± 9.55	69.23 ± 10.85 d	61.16 ± 49.17 d	PT + VRT	C	①
23	Dai et al. (39)	2020	China	41–41	62.03 ± 7.11	61.33 ± 7.50	15.09 ± 4.17 d	15.23 ± 4.88 d	PT	C	①②
24	Liu et al. (40)	2019	China	21–21	40–79		1–3 m		PT	C	②
25	Chang et al. (3)	2024	China	50–50	60.58 ± 4.7	61.04 ± 4.69	59.64 ± 9.15 d	58.79 ± 8.74 d	PT + AM	PT	①②
26	Dai et al. (41)	2019	China	58–58	59.24 ± 6.92	58.82 ± 7.17	24.53 ± 2.65 d	25.23 ± 3.14 d	PT + AFOT	PT	①②
27	Liu et al. (42)	2023	China	20–20	54.21 ± 12.25	54.06 ± 12.79	23.5 ± 4.7 d	21.2 ± 4.2 d	PT + RT2	PT	①②
28	Du and Li (43)	2019	China	40–40	62.41 ± 7.28	62.38 ± 7.25	5.3 ± 2.64 d	5.27 ± 2.61 d	PT + TOT	C	①②
29	Fang et al. (44)	2013	China	32–32					PT	C	②
30	Le et al. (45)	2019	China	15–15	48.8 ± 6.8	50.2 ± 6.3	57.3 ± 9.4 d	58.4 ± 9.9 d	PT	C	①
31	Guan (46)	2020	China	24–24	48–76		7–21 d		PT	C	①
32	Li et al. (47)	2023	China	46–46	59.63 ± 5.37	59.37 ± 6.34	28.64 ± 5.54 d	29.64 ± 5.42 d	PT	C	①②
33	He et al. (48)	2017	China	42–42	61.26 ± 8.85	61 ± 8.64	2.17 ± 0.99 w	2.35 ± 0.96 w	PT + AM	C	②
34	Jiang (49)	2020	China	30–30	63.15 ± 9.85	63.62 ± 10.03	6.75 ± 1.87 m	6.12 ± 1.37 m	PT+C	C	①②
35	Jiao (50)	2024	China	52–52	58.23 ± 3.40	58.12 ± 3.45	3.50 ± 0.49 m	3.45 ± 0.53 m	PT	C	①③
36	Pan et al. (51)	2011	China	31–30	57.31 ± 7.54	54.68 ± 8.21	22.9 ± 13.2 d	24.1 ± 12.9 d	PT	C	①②
37	Zhou YJ (52)	2020	China	31–31	64.39 ± 8.69	65.31 ± 8.41	1.35 ± 0.2 m	1.30 ± 0.2 m	PT	C	②
38	Li (53)	2021	China	49–49	53.33 ± 4.63	53.18 ± 4.58			PT+RRT	C	①②
39	Li (54)	2020	China	50–50	58.2 ± 16.5	54.6 ± 13.7	14.6 ± 1.5 d	13.9 ± 1.3 d	PT	C	①
40	Li DQ (55)	2021	China	30–30	50.41 ± 4.62	52.13 ± 5.04	16.73 ± 4.27 d	18.21 ± 4.69 d	PT+CST	C	①②
41	Li and Li (56)	2019	China	39–39	62.60 ± 9.93	62.03 ± 8.94	1.92 ± 0.60 m	2.05 ± 0.32 m	PT+AM	C	①②③
42	Liang and Sha (57)	2018	China	39–39	61.64 ± 8.52	60.44 ± 8.36	34.12 ± 5.68 d	35.62 ± 5.49 d	PT	C	①②
43	Liu MJ (58)	2017	China	20–20	46.5 ± 13.7	46.7 ± 17.5	8.7 ± 5.7 w	10.5 ± 6.4 w	PT	C	①
44	Lv ZX (59)	2024	China	36–36	61.02 ± 2.75	60.88 ± 2.76			PT	C	①②
45	Zhang (60)	2021	China	32–32	55.21 ± 4.81	54.89 ± 4.22	40.08 ± 5.84 d	39.98 ± 5.79 d	PT+ST	C	①
46	Wu XJ (61)	2020	China	23–22-23	55.67 ± 5.02	53.45 ± 8.32	82.32 ± 9.06 d	82.60 ± 4.17 d	PT+RRT	PT	①②
						56.12 ± 9.10		84.12 ± 7.56 d		C	
47	Wang (62)	2018	China	20–20	64.1 ± 4.8	61.3 ± 5.7			PT+AM	C	①②③
48	Wu XJ (63)	2023	China	24–25	63.00 ± 7.87	60.08 ± 7.78	127.25 ± 24.26 d	128.96 ± 22.14 d	PT	C	②
49	Liu (64)	2017	China	49–49	60.96 ± 3.66	61.16 ± 3.10	35.98 ± 8.44 d	35.86 ± 8.70 d	PT	C	①
50	Li XJ (65)	2016	China	25–25	58.50 ± 17.41	54.50 ± 12.69	15.0 ± 1.5 d	14.0 ± 2.0 d	PT	C	①
51	Lyu YL (66)	2024	China	49–49	63.75 ± 6.76	62.67 ± 6.71			PT+AM	PT	①②
52	Xin YF (67)	2020	China	30–30	58.13 ± 5.07	57.3 ± 4.83	44.73 ± 4.22 d	44.1 ± 4.61 d	PT	C	①③
53	Kim (68)	2015	Korea	10–10	65.9 ± 6.2	64.1 ± 3.6	11.3 ± 1.1 m	12.3 ± 1.3 m	PT	C	①③
54	Hwangbo and Don Kim (69)	2016	Korea	15–15	59.4 ± 9.1	55.9 ± 9.8	11.2 ± 3.6 m	10.9 ± 3.5 m	PT	C	①
55	Choi (70)	2013	Korea	15–15	53.4 ± 9.5	54.1 ± 8.6			PT+KT	C	①
56	Chae (7)	2017	Korea	15–15	58.27 ± 13.11	55.07 ± 13.83	13.53 ± 5.13 m	15.27 ± 4.18 m	PT	C	①③
57	Momna Asghar (71)	2021	Pakistan	30–30	53.63 ± 9.50	53.90 ± 9.43	25.87 ± 12.23 m	23.37 ± 12.99 m	PT	C	①
58	Yeo (72)	2023	Korea	13–13-13	44.85 ± 15.63	56.92 ± 8.95	15.15 ± 4.67 m	14.92 ± 5.42 m	PT+VFT	PT	①③
						51.54 ± 12.74		16.85 ± 4.74 m		C	
59	Park and Oh (73)	2020	Korea	21–21	67.43 ± 4.74	67.57 ± 3.28	13.00 ± 2.68 m	13.48 ± 2.82 m	PT	C	①
60	Lee (74)	2015	Korea	18–18			11.61 ± 2.28 m	11.5 ± 1.58 m	PT+MIT	PT	③
61	Kim and Kang (75)	2018	Korea	12–11	60.75 ± 3.11	60.64 ± 3.41	19.67 ± 3.89 m	19.09 ± 3.86 m	PT	C	③
62	Mao (12)	2022	China	30–30	60.00 ± 9.10	61.10 ± 7.88	97.73 ± 21.23 d	88.13 ± 21.55 d	PT+RRT	C	①②
63	Shim (13)	2020	Korea	17–17	59.65 ± 16.52	56.00 ± 15.61	11.59 ± 5.90 m	13.88 ± 5.51 m	PT+ES	PT	①
64	Kim and Kim (14)	2022	Korea	15–15	53.27 ± 10.12	54.13 ± 9.35	8.53 ± 2.13 m	8.80 ± 2.01 m	PT+TS	C	①

Berg Balance Scale (BBS); Fugl-Meyer assessment-lower extremity (FMA-LE); Timed Up-and-Go Test (TUGT).

CG: control group; TG: Treatment group; PT: Proprioceptive Training; DTT: Dual Task Training; CKCE: Closed Kinematic Chain Exercise; RT1: Resistance Training; CST: Core Stability Training; AM: Acupuncture and Moxibustion; VFT: Visual Feedback Training; ES: Electric Stimulation; KT: Kinesio taping; RT2: Rope Therapy; TOT: Task Oriented Training; RRT: Rehabilitation Robot Therapy; MRP: Motor Relearning Programme; ST: Shock Therapy; SBT: Static Balance Training; VRT: Virtual Reality Balance Training; TS: Thermal Stimulation; AFOT: Ankle-Foot Orthosis Therapy; MIT: Motor Imagery Training; C: Conventional Rehabilitation.

### 3.3 Quality evaluation of included studies

**Figure 2:**
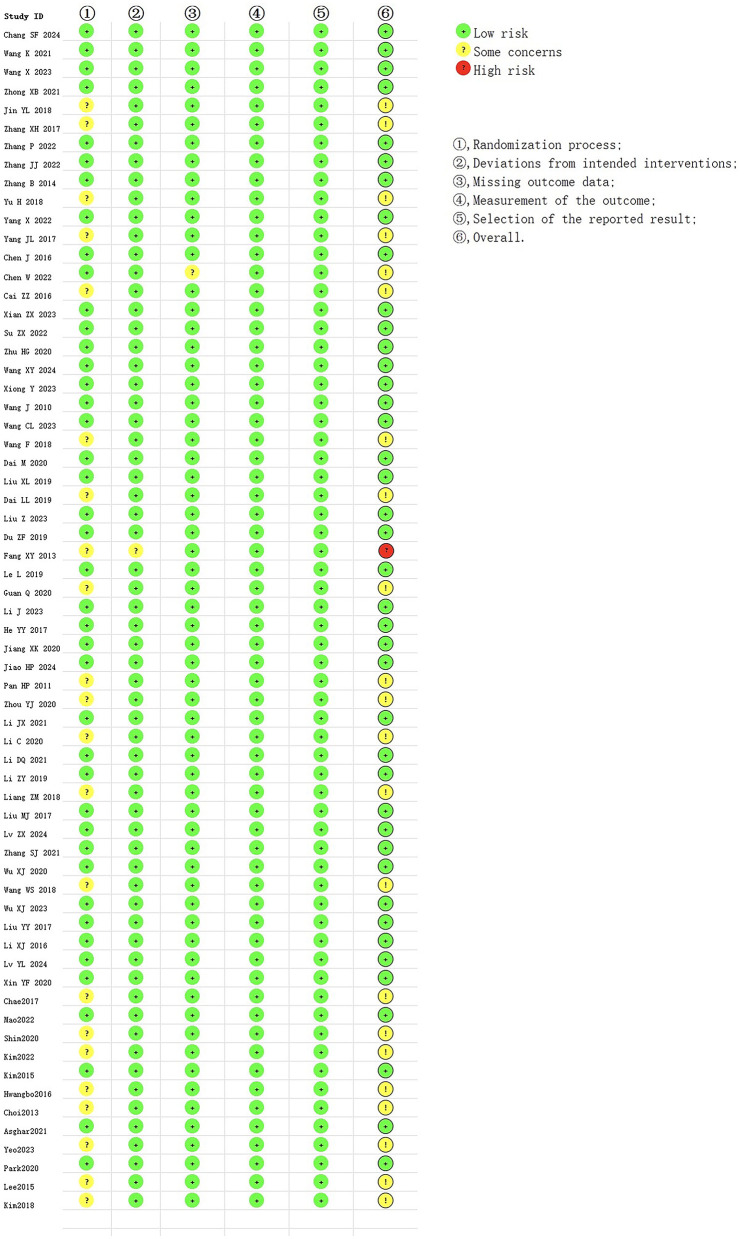
Quality assessment of included studies.

Forty-one studies (3, 12, 17–19, 22–24, 26, 28, 31–37, 39, 40, 42, 43, 45, 47–50, 53, 55, 56, 58–61, 63–68, 71, 73) had a low risk of bias; 22 studies (7, 13, 14, 20, 21, 25, 27, 29, 30, 38, 41, 46, 51, 52, 54, 57, 62, 69, 70, 72, 74, 75) had a moderate risk of bias; and one study (44) had a high risk of bias. In terms of the randomization, 22 studies had a moderate risk of bias and 42 studies had a low risk of bias. In terms of whether the intervention measures deviated from the expected, one study had a moderate risk of bias and 63 studies had a low risk of bias. For missing result data, one study had a moderate risk of bias and 63 studies had a low risk of bias. In terms of outcome measurement, 64 studies had a low risk of bias. In terms of selective reporting, 64 studies had a low risk of bias. In summary, the moderate risk of bias in the randomization domain was the main source of bias in the included studies. The risk of bias of the included studies is depicted in Figure 2.

### 3.4 Network meta-analysis

**Figure 3:**
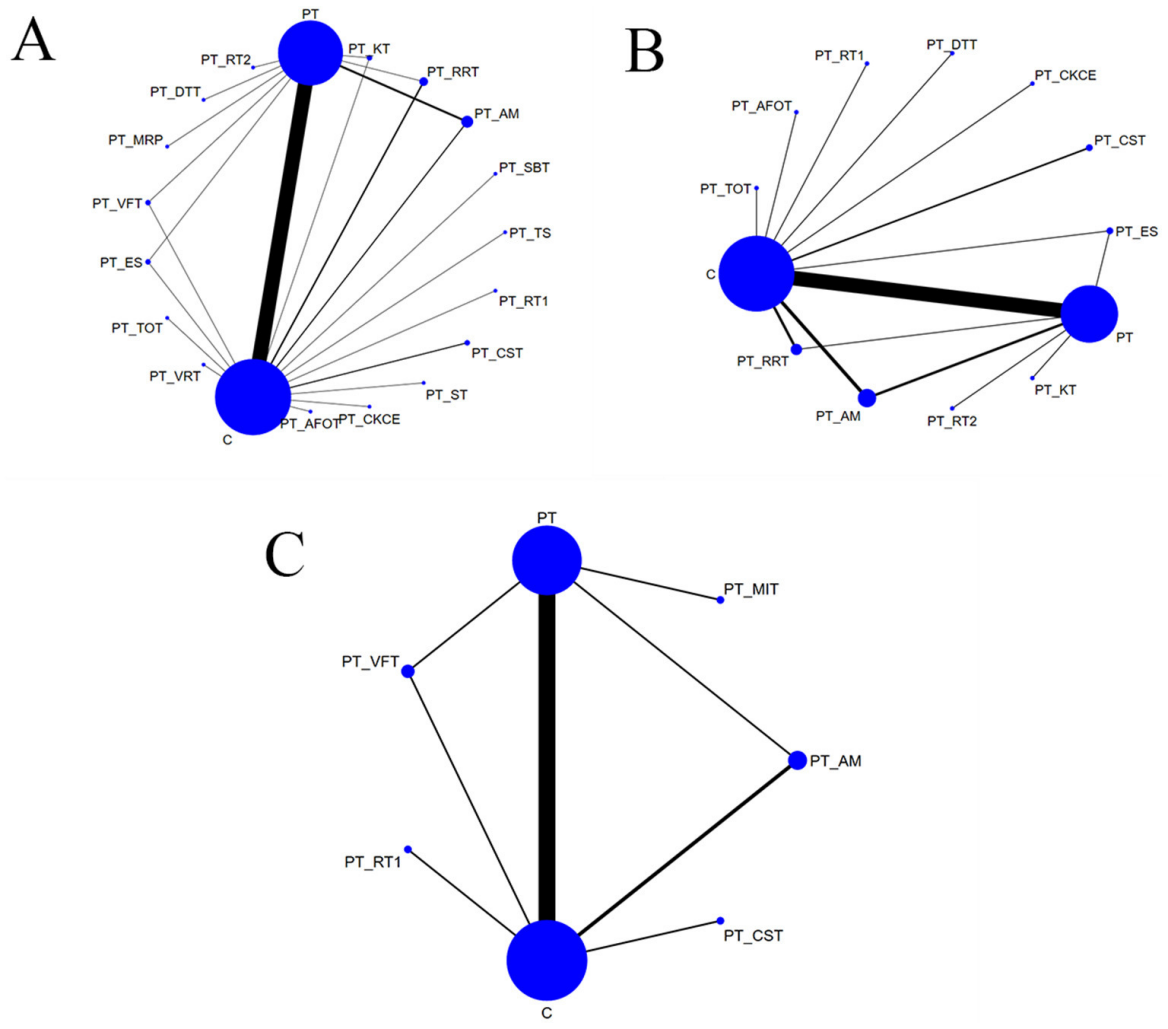
**(A)** Network diagram of BBS; **(B)** network diagram of FMA-LE; **(C)** network diagram of TUGT.

The network diagrams corresponding to the three outcome measures are shown in Figure 3. The size of each node was directly proportional to the sample size involved in each intervention, and the thickness of the lines between nodes represented the number of studies comparing the interventions connected by the lines. Larger nodes indicate a greater sample size, while thicker lines represent a higher number of studies.

#### 3.4.1 Berg Balance Scale

A total of 53 studies (3, 7, 12–14, 17–22, 24–27, 29–33, 35, 37–39, 41–43, 45–47, 49–51, 53–62, 64–73) reported BBS scores, involving 19 rehabilitation interventions: C (conventional rehabilitation), PT (proprioceptive training), PT + DTT (dual-task training), PT + RT1 (resistance training), PT + CST (core stability training), PT + AM (acupuncture and moxibustion), PT + MRP (motor relearning programme), PT + SBT (static balance training), PT + ES (electrical stimulation), PT + KT (kinesio taping), PT + VRT (virtual reality training), PT + RT2 (rope therapy), PT + TOT (task-oriented training), PT + RRT (robotic rehabilitation therapy), PT + ST (shock therapy), PT + VFT (visual feedback training), PT + TS (thermal stimulation), and PT + AFOT (ankle-foot orthosis treatment), PT + CKCE (closed kinematic chain exercises). Direct comparisons were conducted between PT + VFT, PT + AM, PT + KT, PT + RRT, PT + ES and PT as well as C. Additionally, PT was directly compared with PT + MRP, PT + DTT, PT + RT2, and C, while C was directly compared with PT + TOT, PT + VRT, PT + SBT, PT + TS, PT + RT1, PT + CST, PT + ST, PT + CKCE, PT + AFOT, and PT. The results are provided in Figure 3A.

The league table for BBS is shown in the upper right part of Table 2. The data in the league table were derived from pairwise comparisons between various interventions. In terms of improving BBS scores, the following interventions were found to be more effective than the control (C): PT (MD = 4.94, 95% CI: 3.18–6.71), PT + AM (MD = 9.92, 95% CI: 5.81–14.02), PT + CKCE (MD = 10.89, 95% CI: 1.32–20.45), PT + DTT (MD = 10.17, 95% CI: 0.49–19.84), PT + MRP (MD = 12.33, 95% CI: 2.59–22.12), PT + RRT (MD = 9.56, 95% CI: 4.27–14.9), and PT + VFT (MD = 9.7, 95% CI: 0.55–18.89). Additionally, PT + AM (MD = 4.98, 95% CI: 1.02–8.93) was more effective than PT alone, with all differences being statistically significant (*P* < 0.05).

**Table 2:** League table of BBS and FMA-LE.

**FMA-LE**	**MD 95%CI**																			**BBS**
	**C**	4.94 (3.18, 6.71)	7.65 (−1.82, 17.04)	9.92 (5.81, 14.02)	10.89 (1.32, 20.45)	5.61 (−1.29, 12.55)	10.17 (0.49, 19.84)	12.33 (2.59, 22.12)	3.42 (−3.77, 10.66)	5.46 (−1.27, 12.2)	9.56 (4.27, 14.9)	7.85 (−1.7, 17.42)	8.48 (−1.2, 18.16)	9.04 (−0.37, 18.54)	8.8 (−0.6, 18.23)	7.58 (−2.03, 17.15)	1.94 (−7.48, 11.27)	9.7 (0.55, 18.89)	3.49 (−7.65, 14.62)	
	−4.51 (−5.72, −3.33)	**PT**	2.72 (−6.92, 12.26)	4.98 (1.02, 8.93)	5.95 (−3.77, 15.66)	0.66 (−6.46, 7.81)	5.23 (−4.31, 14.78)	7.4 (−2.18, 17.01)	−1.52 (−8.75, 5.78)	0.52 (−6.21, 7.26)	4.62 (−0.85, 10.14)	2.91 (−6.83, 12.64)	3.53 (−5.95, 13.05)	4.1 (−5.49, 13.73)	3.86 (−5.7, 13.45)	2.64 (−7.13, 12.34)	−3.02 (−12.56, 6.5)	4.78 (−4.4, 13.93)	−1.44 (−12.77, 9.83)	
	−3.4 (−8.29, 1.47)	1.11 (−3.89, 6.15)	**PT** **+** **AFOT**	2.27 (−7.99, 12.58)	3.22 (−10.14, 16.7)	−2.06 (−13.69, 9.67)	2.53 (−10.98, 16.03)	4.67 (−8.81, 18.25)	−4.24 (−16.04, 7.64)	−2.2 (−13.77, 9.4)	1.92 (−8.89, 12.78)	0.18 (−13.21, 13.64)	0.81 (−12.62, 14.4)	−0.09 (−13.46, 13.38)	1.16 (−12.15, 14.49)	−0.09 (−13.46, 13.38)	−5.71 (−19, 7.57)	2.06 (−11.08, 15.24)	−4.16 (−18.82, 10.47)	
	−6.73 (−8.71, −4.7)	−2.22 (−4.22, −0.15)	−3.34 (−8.58, 1.98)	**PT** **+** **AM**	0.96 (−9.41, 11.36)	−4.31 (−12.35, 3.75)	0.26 (−10.06, 10.54)	2.42 (−7.93, 12.85)	−6.5 (−14.66, 1.69)	−4.46 (−12.24, 3.28)	−0.36 (−6.96, 6.31)	−2.07 (−12.48, 8.31)	−1.45 (−11.74, 8.87)	−0.87 (−11.14, 9.43)	−1.11 (−11.34, 9.14)	−2.33 (−12.8, 8.11)	−7.98 (−18.22, 2.22)	−0.2 (−10.14, 9.68)	−6.42 (−18.28, 5.41)	
	−9.98 (−14.97, −5.01)	−5.47 (−10.59, −0.33)	−6.59 (−13.52, 0.39)	−3.25 (−8.62, 2.08)	**PT** **+** **CKCE**	−5.26 (−17.08, 6.57)	−0.71 (−14.34, 12.86)	1.43 (−12.2, 15.1)	−7.46 (−19.45, 4.5)	−5.43 (−17.07, 6.22)	−1.29 (−12.29, 9.57)	−3.02 (−16.54, 10.47)	−2.41 (−16.03, 11.24)	−1.81 (−15.2, 11.6)	−2.08 (−15.41, 11.34)	−3.31 (−16.82, 10.2)	−8.94 (−22.34, 4.42)	−1.17 (−14.45, 12.05)	−7.39 (−22.15, 7.35)	
	−3.16 (−7.03, 0.64)	1.35 (−2.68, 5.37)	0.25 (−6.01, 6.41)	3.57 (−0.8, 7.87)	6.82 (0.5, 13.07)	**PT** **+** **CST**	4.57 (−7.37, 16.48)	6.72 (−5.23, 18.71)	−2.2 (−12.11, 7.83)	−0.16 (−9.84, 9.53)	3.95 (−4.77, 12.63)	2.22 (−9.58, 14.04)	2.86 (−9, 14.76)	3.45 (−8.27, 15.12)	3.18 (−8.48, 14.91)	1.97 (−9.93, 13.79)	−3.67 (−15.37, 7.99)	4.09 (−7.41, 15.64)	−2.11 (−15.25, 10.99)	
	−2.29 (−7.23, 2.69)	2.22 (−2.85, 7.36)	1.1 (−5.83, 8.04)	4.44 (−0.91, 9.76)	7.69 (0.71, 14.7)	0.86 (−5.35, 7.17)	**PT** **+** **DTT**	2.16 (−11.39, 15.7)	−6.75 (−18.67, 5.25)	−4.7 (−16.42, 6.96)	−0.62 (−11.59, 10.43)	−2.32 (−15.92, 11.24)	−1.71 (−15.14, 11.82)	−1.12 (−14.64, 12.39)	−1.38 (−14.88, 12.23)	−2.59 (−16.24, 11.11)	−8.21 (−21.76, 5.27)	−0.49 (−13.68, 12.82)	−6.67 (−21.5, 8.04)	
								**PT** **+** **MRP**	−8.91 (−20.89, 3.12)	−6.86 (−18.61, 4.8)	−2.77 (−13.85, 8.23)	−4.49 (−18.18, 9.15)	−3.87 (−17.42, 9.67)	−3.29 (−16.81, 10.33)	−3.52 (−17.11, 10.03)	−4.76 (−18.42, 8.9)	−10.38 (−23.95, 3.13)	−2.63 (−15.82, 10.57)	−8.84 (−23.74, 5.91)	
	−3.98 (−8.16, −0.07)	0.52 (−3.68, 4.48)	−0.58 (−7.07, 5.59)	2.75 (−1.9, 7.06)	6 (−0.59, 12.25)	−0.82 (−6.53, 4.63)	−1.69 (−8.23, 4.55)		**PT** **+** **ES**	2.03 (−7.76, 11.81)	6.15 (−2.83, 15.02)	4.44 (−7.59, 16.4)	5.07 (−6.99, 17.03)	5.62 (−6.27, 17.49)	5.39 (−6.5, 17.26)	4.17 (−7.86, 16.13)	−1.48 (−13.34, 10.32)	6.29 (−5.37, 17.85)	0.07 (−13.25, 13.28)	
	−8.9 (−13.89, −3.94)	−4.4 (−9.23, 0.44)	−5.5 (−12.44, 1.45)	−2.18 (−7.44, 3.04)	1.08 (−5.98, 8.1)	−5.75 (−11.97, 0.55)	−6.61 (−13.64, 0.38)		−4.92 (−11.09, 1.57)	**PT** **+** **KT**	4.09 (−4.37, 12.66)	2.38 (−9.28, 14.04)	3.01 (−8.66, 14.69)	3.59 (−7.99, 15.26)	3.34 (−8.21, 14.93)	2.12 (−9.63, 13.87)	−3.54 (−15.08, 8)	4.25 (−7.02, 15.57)	−1.98 (−14.97, 11.09)	
	−5.66 (−8.43, −2.96)	−1.15 (−4.07, 1.74)	−2.26 (−7.88, 3.32)	1.07 (−2.33, 4.39)	4.32 (−1.38, 9.97)	−2.5 (−7.24, 2.21)	−3.37 (−9.11, 2.26)		−1.68 (−6.43, 3.28)	3.24 (−2.42, 8.86)	**PT** **+** **RRT**	−1.72 (−12.68, 9.2)	−1.08 (−12.05, 9.84)	−0.51 (−11.32, 10.35)	−0.73 (−11.61, 10.03)	−1.98 (−12.98, 8.93)	−7.62 (−18.42, 3.15)	0.16 (−10.42, 10.72)	−6.07 (−18.4, 6.26)	
	−9.07 (−14.07, −4.09)	−4.57 (−9.67, 0.58)	−5.66 (−12.63, 1.3)	−2.33 (−7.73, 3.02)	0.9 (−6.11, 7.95)	−5.91 (−12.17, 0.45)	−6.78 (−13.81, 0.27)		−5.09 (−11.35, 1.49)	−0.16 (−7.18, 6.88)	−3.4 (−9.08, 2.35)	**PT** **+** **RT1**	0.61 (−12.97, 14.27)	1.23 (−12.24, 14.67)	0.96 (−12.42, 14.38)	−0.28 (−13.84, 13.23)	−5.91 (−19.34, 7.46)	1.86 (−11.36, 15.09)	−4.37 (−19.05, 10.33)	
	−6.34 (−11.7, −1.04)	−1.83 (−7.03, 3.36)	−2.95 (−10.16, 4.24)	0.38 (−5.23, 5.92)	3.64 (−3.66, 10.9)	−3.19 (−9.72, 3.4)	−4.05 (−11.36, 3.19)		−2.35 (−8.83, 4.4)	2.56 (−4.55, 9.62)	−0.68 (−6.62, 5.28)	2.73 (−4.63, 10)	**PT** **+** **RT2**	0.58 (−12.94, 14.13)	0.33 (−13.17, 13.81)	−0.89 (−14.6, 12.69)	−6.54 (−20.03, 6.95)	1.24 (−12.02, 14.47)	−5.01 (−19.77, 9.77)	
	−6.73 (−11.59, −1.89)	−2.22 (−7.18, 2.79)	−3.33 (−10.21, 3.58)	0 (−5.26, 5.24)	3.25 (−3.7, 10.2)	−3.56 (−9.74, 2.65)	−4.45 (−11.39, 2.5)		−2.76 (−8.86, 3.71)	2.17 (−4.77, 9.15)	−1.07 (−6.62, 4.54)	2.33 (−4.61, 9.29)	−0.38 (−7.56, 6.84)	**PT** **+** **TOT**	−0.25 (−13.65, 13.09)	−1.49 (−14.94, 11.94)	−7.13 (−20.44, 6.17)	0.66 (−12.54, 13.82)	−5.56 (−20.24, 9)	
															**PT** **+** **SBT**	−1.21 (−14.67, 12.17)	−6.85 (−20.17, 6.46)	0.91 (−12.23, 13.94)	−5.31 (−19.96, 9.27)	
																**PT** **+** **ST**	−5.66 (−19.02, 7.86)	2.12 (−11.15, 15.4)	−4.1 (−18.8, 10.69)	
																	**PT** **+** **TS**	7.78 (−5.35, 20.93)	1.55 (−13.03, 16.17)	
																		**PT** **+** **VFT**	−6.21 (−20.64, 8.19)	
																			**PT** **+** **VRT**	

BBS, Berg Balance Scale; FMA-LE, Fugl-Meyer assessment-lower extremity; PT, Proprioceptive Training; DTT, Dual Task Training; CKCE, Closed Kinematic Chain Exercise; RT1, Resistance Training; CST, Core Stability Training; AM, Acupuncture and Moxibustion; VFT, Visual Feedback Training; ES, Electric Stimulation; KT, Kinesio taping; RT2, Rope Therapy; TOT, Task Oriented Training; RRT, Rehabilitation Robot Therapy; MRP, Motor Relearning Programme; ST, Shock Therapy; SBT, Static Balance Training; VRT, Virtual Reality Balance Training; TS, Thermal Stimulation; AFOT, Ankle-Foot Orthosis Therapy; C, Conventional Rehabilitation.

**Figure 4:**
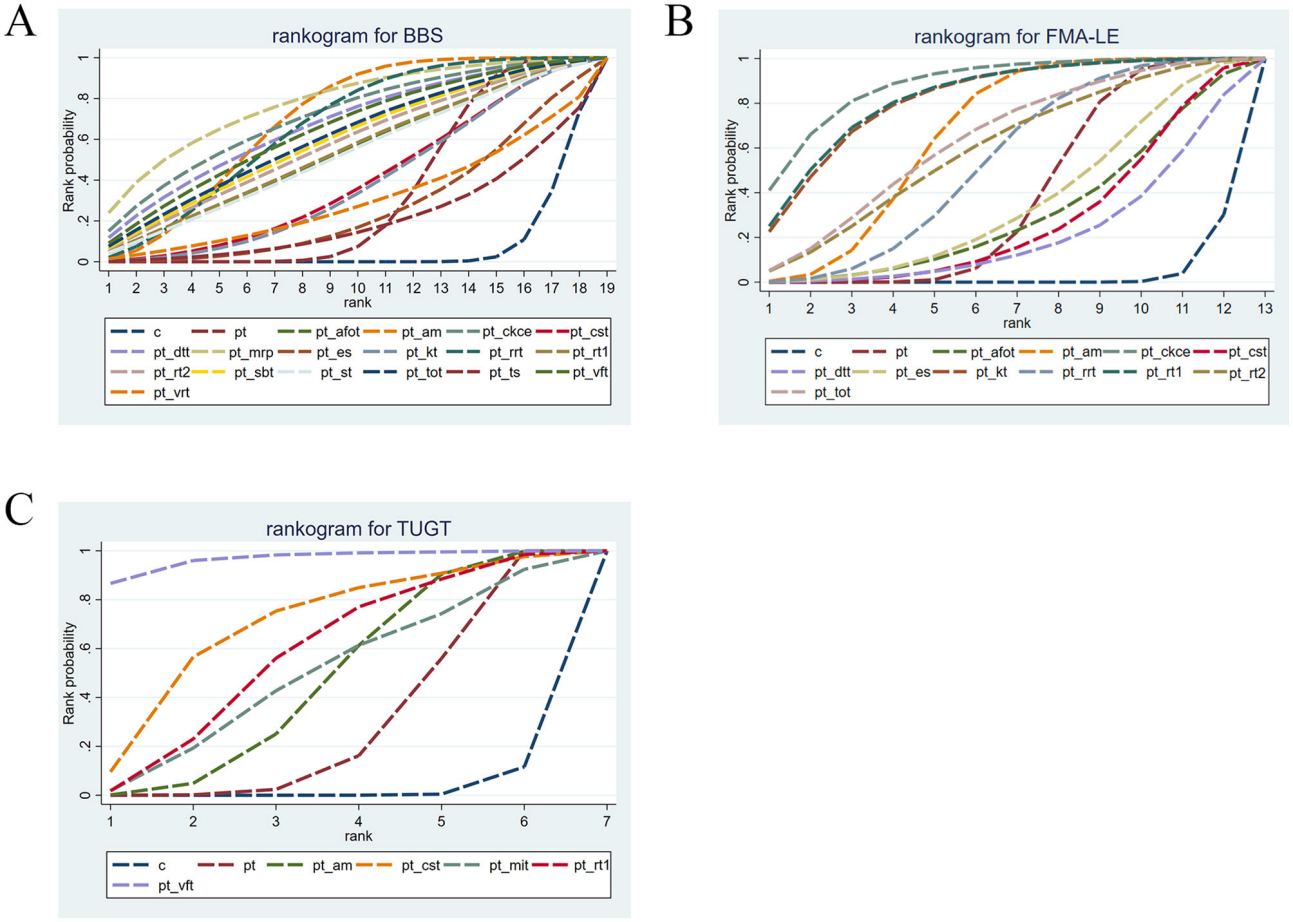
**(A)** Cumulative probability ranking of BBS; **(B)** cumulative probability ranking of FMA-LE; **(C)** cumulative probability ranking of TUGT.

The cumulative probability ranking for BBS is shown in Table 4. After comparing all interventions, the cumulative probability ranking was calculated to determine the effectiveness of each intervention, thereby identifying the optimal intervention. According to the SUCRA ranking, the top three optimal interventions were PT+MRP (77.94%), PT + CKCE (70.94%), and PT + AM (69.52%). The cumulative probability curve for BBS is shown in Figure 4A. PT + MRP was identified as the optimal treatment regimen for improving lower limb balance in stroke patients.

#### 3.4.2 Fugl-Meyer Assessment for Lower Extremity

A total of 37 studies (3, 12, 17–20, 22–24, 26–28, 31, 34–37, 39–44, 47–49, 51–53, 55–57, 59, 61–63, 66) reported FMA-LE scores, involving 13 rehabilitation interventions: C (conventional rehabilitation), PT (proprioceptive training), PT + DTT (dual-task training), PT + CKCE (closed kinematic chain exercises), PT + RT1 (resistance training), PT + CST (core stability training), PT + AM (acupuncture and moxibustion), PT + ES (electrical stimulation), PT + KT (kinesio taping), PT + RT2 (rope therapy), PT + TOT(task-oriented training), PT + RRT (robotic rehabilitation therapy), and PT + AFOT (ankle-foot orthosis treatment). Direct comparisons were made between PT + ES, PT + AM, PT + RRT, and PT as well as C. PT was also directly compared with PT + RT2, PT + KT, and C, while C was directly compared with PT + CKCE, PT + CST, PT + DTT, PT + RT1, PT + AFOT, PT + TOT, and PT. The results are shown in Figure 3B.

The league table for FMA-LE is shown in the lower left part of Table 2. In terms of improving FMA-LE scores, the following interventions were found to be more effective than the control (C): PT (MD = 4.51, 95% CI: 3.33–5.72), PT + AM (MD = 6.73, 95% CI: 4.7–8.71), PT + CKCE (MD = 9.98, 95% CI: 5.01–14.97), PT + ES (MD = 3.98, 95% CI: 0.07–8.16), PT + KT (MD = 8.9, 95% CI: 3.94–13.89), PT + RRT (MD = 5.66, 95% CI: 2.96–8.43), PT + RT1 (MD = 9.07, 95% CI: 4.09–14.07), PT + RT2 (MD = 6.34, 95% CI: 1.04–11.7), and PT + TOT (MD = 6.73, 95% CI: 1.89–11.59). Additionally, PT + AM (MD = 2.22, 95% CI: 0.15–4.22) and PT + CKCE (MD = 5.47, 95% CI: 0.33–10.59) were more effective than PT alone, while PT + CKCE was more effective than PT + CST (MD = −6.82, 95% CI: −13.07 to −0.5) and PT + DTT (MD = −7.69, 95% CI: −14.7 to −0.71), with all differences being statistically significant (*P* < 0.05).

The cumulative probability ranking for FMA-LE is shown in Table 4. According to the SUCRA ranking, the top three optimal interventions were PT + CKCE (88.39%), PT + RT1 (82.7%), and PT + KT (81.88%). The cumulative probability curve for FMA-LE is shown in Figure 4B. PT + CKCE was identified as the optimal treatment regimen for improving lower limb motor function in stroke patients.

#### 3.4.3 Timed Up and Go Test

A total of 15 studies (7, 20, 23–26, 31, 50, 56, 62, 67, 68, 72, 74, 75) reported TUGT times, involving seven rehabilitation interventions: C (conventional rehabilitation), PT (proprioceptive training), PT + RT1 (resistance training), PT + CST (core stability training), PT + AM (acupuncture and moxibustion), PT + VFT (visual feedback training), and PT + MIT (motor imagery training). Direct comparisons were made between PT + VFT, PT + AM and PT as well as C. Additionally, PT was directly compared with PT + MIT, while C was directly compared with PT + CST and PT + RT1. The results are shown in Figure 3C.

The league table for TUGT is shown in Table 3. In terms of reducing TUGT time, the following interventions were found to be more effective than the control (C): PT (MD = −3.07, 95% CI: −4.32 to −1.78), PT + AM (MD = −4.29, 95% CI: −7 to −1.75), PT + CST (MD = −7.73, 95% CI: −15.45 to 0), PT + RT1 (MD = −5.63, 95% CI: −10.47 to −0.55), and PT + VFT (MD = −15.54, 95% CI: −25.1 to −5.86). However, PT alone (MD = −12.47, 95% CI: −22.07 to −2.8) and PT + AM (MD = −11.22, 95% CI: −21.12 to −1.17) were less effective than PT + VFT, with all differences being statistically significant (*P* < 0.05).

**Table 3:** League table of TUGT.

**MD95%CI**
**C**	−3.07 (−4.32, −1.78)	−7.73 (−15.45, 0)	−5.63 (−10.74, −0.55)	−4.29 (−7, −1.75)	−4.8 (−11.43, 1.83)	−15.54 (−25.1, −5.86)	TUGT
	**PT**	−4.67 (−12.5, 3.16)	−2.57 (−7.83, 2.66)	−1.22 (−3.97, 1.32)	−1.73 (−8.25, 4.78)	−12.47 (−22.07, −2.8)	
		**PT** **+** **CST**	2.08 (−7.16, 11.36)	−3.42 (−11.53, 4.77)	2.95 (−7.29, 13.06)	−7.78 (−20.13, 4.54)	
			**PT** **+** **RT1**	−1.34 (−6.99, 4.44)	−0.83 (−9.17, 7.53)	−9.87 (−20.7, 1.09)	
				**PT** **+** **AM**	−0.49 (−7.47, 6.57)	−11.22 (−21.12, −1.17)	
					**PT** **+** **MIT**	−10.71 (−22.36, 0.97)	
						**PT** **+** **VFT**	

TUGT, Timed Up-and-Go Test; PT, Proprioceptive Training; RT1, Resistance Training; CST, Core Stability Training; AM, Acupuncture and Moxibustion; MIT, Motor Imagery Training; VFT, Visual Feedback Training; C, Conventional Rehabilitation.

The cumulative probability ranking for TUGT is shown in Table 4. Based on the SUCRA ranking for reducing TUGT time, the top three optimal interventions were PT + VFT (96.61%), PT + CST (69.15%), and PT + RT1 (57.5%). The cumulative probability curve for TUGT is shown in Figure 4C. PT + VFT was identified as one of the optimal treatments for improving walking ability in stroke patients.

**Table 4:** Cumulative probability ranking table.

**Intervention**	**BBS**	**FMA-LE**	**TUGT**
C	6.73	2.87	2.01
PT	32.57	38.17	29.11
PT_AFOT	52.81	30.31	0
PT_AM	69.52	66.28	46.94
PT_CKCE	70.94	88.39	0
PT_CST	39.63	26.89	69.15
PT_DTT	67.16	21.12	0
PT_MRP	77.94	0	0
PT_ES	26.57	35.18	0
PT_KT	38.5	81.88	0
PT_RRT	66.43	53.33	0
PT_RT1	53.9	82.7	57.5
PT_RT2	57.55	59.41	0
PT_SBT	59.6	0	0
PT_ST	52.31	0	0
PT_TOT	61.02	63.46	0
PT_TS	21.33	0	0
PT_VFT	64.9	0	96.61
PT_VRT	30.53	0	0
PT_MIT	0	0	48.67

①, Berg Balance Scale (BBS); ②, Fugl-Meyer assessment-lower extremity (FMA-LE); ③, Timed Up-and-Go Test (TUGT).

CG, control group; TG, Treatment group; PT, Proprioceptive Training; DTT, Dual Task Training; CKCE, Closed Kinematic Chain Exercise; RT1, Resistance Training; CST, Core Stability Training; AM, Acupuncture and Moxibustion; VFT, Visual Feedback Training; ES, Electric Stimulation; KT, Kinesio taping; RT2, Rope Therapy; TOT, Task Oriented Training; RRT, Rehabilitation Robot Therapy; MRP, Motor Relearning Programme; ST, Shock Therapy; SBT, Static Balance Training; VRT, Virtual Reality Balance Training; TS, Thermal Stimulation; AFOT, Ankle-Foot Orthosis Therapy; MIT, Motor Imagery Training; C. Conventional Rehabilitation; red column: highest cumulative probability ranking; yellow column: second-highest cumulative probability ranking; green column: third-highest cumulative probability ranking.

### 3.5 Cluster analysis

**Figure 5:**
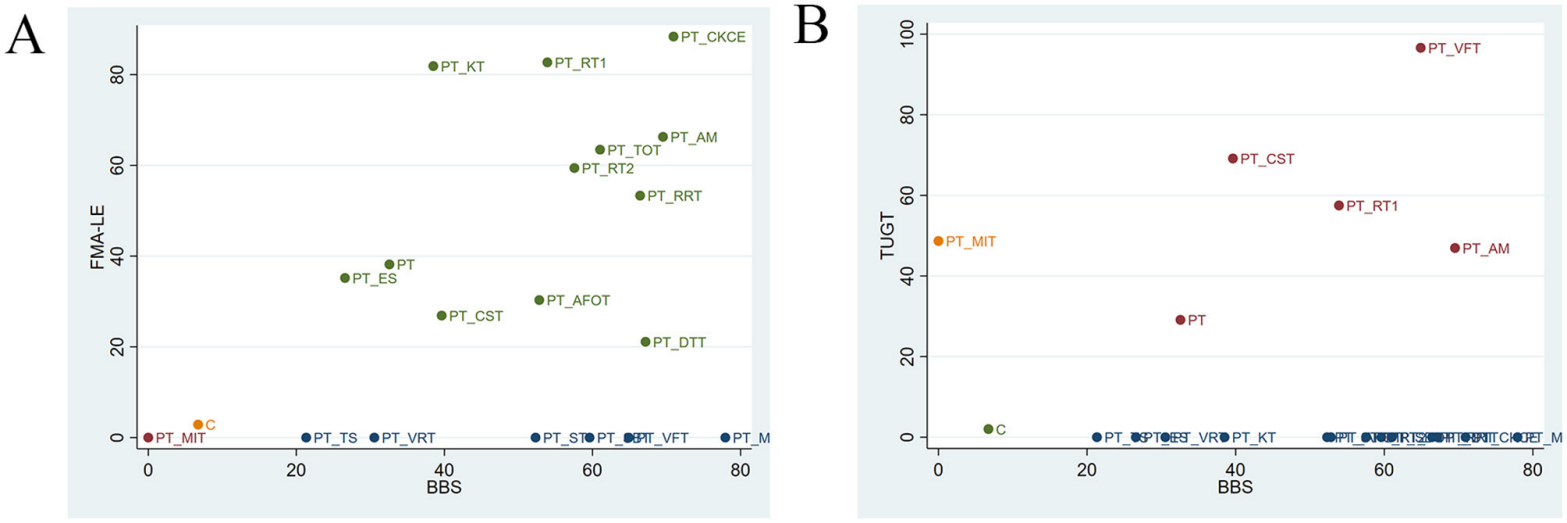
**(A)** Cluster analysis of BBS and FMA-LE; **(B)** cluster analysis of BBS and TUGT.

The cluster analysis is shown in Figure 5. The most effective interventions for improving balance and lower limb motor function in stroke patients were identified to be PT + CKCE (70.94%/88.39%), PT + RT1 (53.9%/82.7%), and PT + AM (69.52%/66.28%), as shown in Figure 5A; PT + VFT (64.9%/96.61%) and PT + RT1 (53.9%/57.5%) were identified to be particularly effective in enhancing balance and walking function in stroke patients, as shown in Figure 5B. PT + CKCE exhibited the best therapeutic effect on balance and lower limb motor function in stroke patients; PT + RT1 demonstrated good efficacy in improving balance, lower limb motor function, and walking ability; PT + VFT, while being the best option for improving balance and walking ability in stroke patients, is less effective in enhancing balance compared to PT + RRT, PT + CKCE, PT + AM, PT + MRP, and PT + DTT. Therefore, the choice of PT + VFT should be made cautiously based on the patient's specific condition. Overall, PT + CKCE and PT + RT1 were identified as the two most effective rehabilitation interventions for treating lower limb dysfunction in stroke patients.

### 3.6 Publication bias

**Figure 6:**
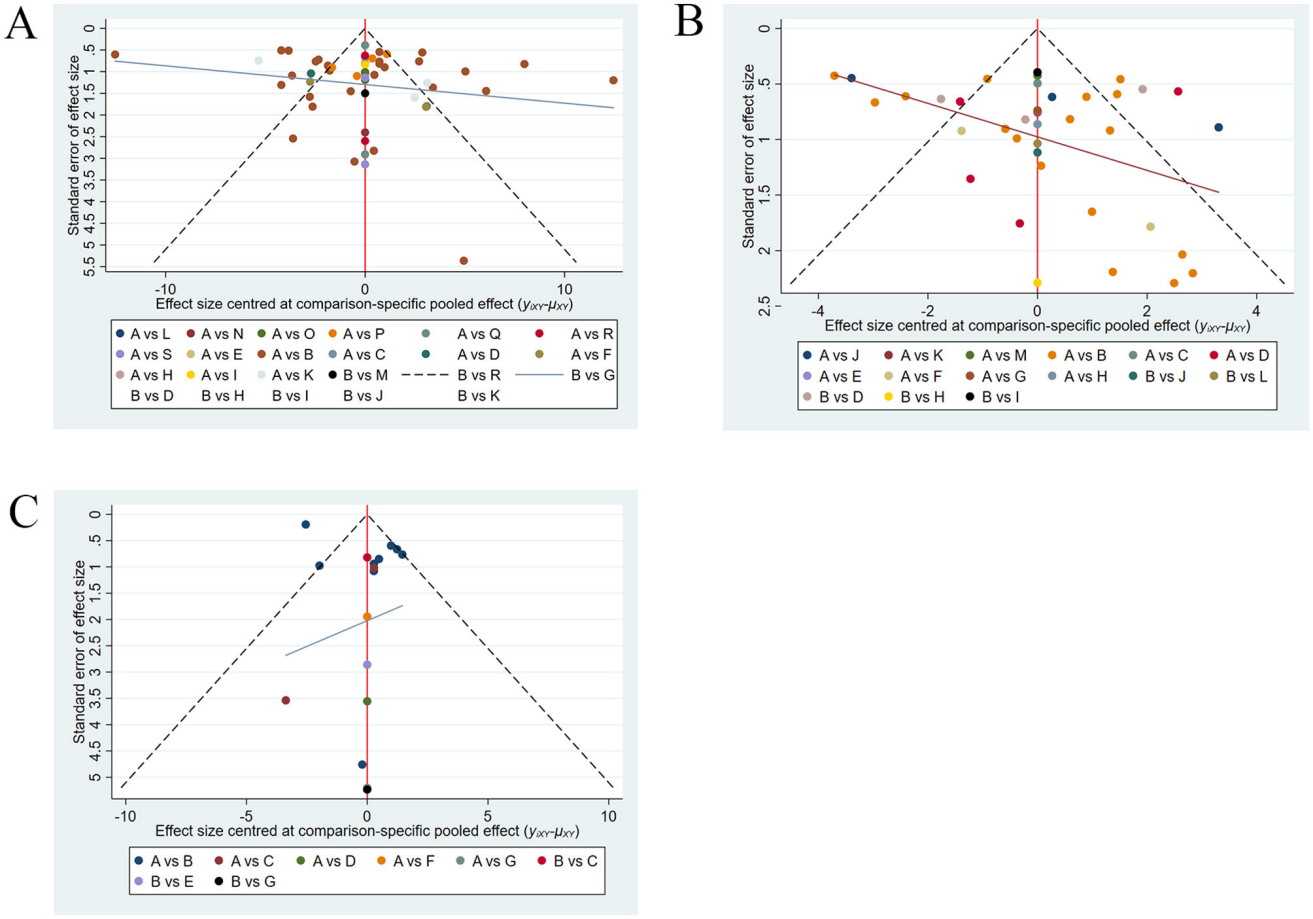
**(A)** Funnel plot of BBS; **(B)** funnel plot of FMA-LE; **(C)** funnel plot of TUGT.

As shown in Figure 6, publication bias was assessed for BBS, FMA-LE, and TUGT. The funnel plots for FMA-LE and TUGT were generally symmetrical, with only a few points falling outside the funnel and a relatively large angle between the reference line and the *X*-axis, suggesting minimal publication bias for these two outcome measures. The funnel plot for BBS was overall symmetrical, with some points falling outside the funnel and a relatively small angle between the reference line and the *X*-axis, suggesting possible publication bias for BBS.

## 4 Discussion

This NMA included 64 RCTs involving 4,084 stroke patients with lower limb dysfunction. Study findings indicated that the optimal intervention for improving BBS scores in stroke patients was PT + motor relearning programme; for FMA-LE scores, PT + closed kinematic chain exercises was the most effective; and for TUGT scores, PT + visual feedback training was the optimal intervention. Overall, PT + closed kinematic chain exercises and PT + resistance training were identified as the two most effective rehabilitation interventions for treating lower limb dysfunction in stroke patients.

BBS score is reflective of the balance ability of stroke patients. The optimal rehabilitation intervention for restoring lower limb balance function in stroke patients was identified to be PT + motor relearning programme. Proprioception helps the body perceive spatial positions, and PT improves neuromuscular control, enhancing postural stability and balance ability (76). In recent years, motor relearning programme has gained significant attention from clinical rehabilitation therapists. This method, proposed by Australian physiotherapists, is a movement therapy based on the neuroplasticity of the central nervous system. It underscores the importance of early rehabilitation in patients with functional impairments, encouraging active patient participation and repetitive training. Under the guidance of professional therapists, it aims to restore the lost lower limb function in stroke patients as early as possible (77). The rehabilitative effect of motor relearning programme on balance function in stroke patients is grounded in the theoretical basis of balance function reorganization following central nervous system injury. This method views the restoration of balance function as a task-oriented process, emphasizing active patient participation and the integration of balance tasks into real-life environments. It focuses on functionality and practicality, re-educating and retraining patients to continuously correct faulty movements and address deficiencies, thereby facilitating the early recovery of balance function (78). Implementing motor relearning programme based on PT is more conducive to promoting the reshaping of the central nervous system in patients with lower limb dysfunction, thereby more efficiently enhancing the recovery of their balance abilities.

FMA-LE score is reflective of the lower limb motor function of stroke patients. The optimal rehabilitation intervention for restoring lower limb motor function in stroke patients was identified to be PT + closed kinematic chain exercises. The recovery of lower limb motor function in stroke patients requires both the enhancement of lower limb muscle strength and the strengthening of proprioceptive input. Therefore, it is evident that PT is an effective method to improve proprioception in the affected limb and promote the recovery of lower limb motor function (12). Closed kinematic chain exercises is a commonly used rehabilitation training method characterized by specific movements with the distal part of the body fixed (79). This method has been proven to enhance lower limb motor function in stroke patients with hemiplegia (80). On one hand, closed kinematic chain exercises involves coordinated linear movements of the three major joints, aligning with the principle of neural development from the hip to the foot. The closed-chain model drives repetitive flexion and extension of the joints, requiring the recruitment of more motor neuron signals for simultaneous contraction of agonist and antagonist muscles. This approach is beneficial for improving muscle strength in the affected limb and enhancing coordination between muscles. On the other hand, post-stroke motor dysfunction is associated with reduced proprioceptive input. Closed kinematic chain exercises, through repetitive training of the three major joints, stimulates joint and position sense, creating conditions for the recovery of lower limb proprioception, and enhances neuromuscular control of the lower limb (81, 82). Closed kinematic chain exercises can effectively improve muscle strength in the affected limb of stroke patients, and when combined with PT for the restoration of proprioception, it can lead to better neuromuscular control of the lower limb. This mechanism is likely the reason why PT+ closed kinematic chain exercises is more effective in improving lower limb motor function in stroke patients.

TUGT score is reflective of the walking ability of stroke patients. The optimal rehabilitation intervention for restoring walking ability in stroke patients was identified to be PT+ visual feedback training. Research has shown that PT is an effective method for enhancing walking ability in stroke patients. The key to improving gait through PT lies in enhancing control of the trunk, pelvis, and lower limbs. During the stance phase, therapists apply compression to the patient's pelvis, and during the swing phase, they utilize traction reflexes on the pelvis. This approach promotes coordinated muscle movement between the lower limbs and trunk, thereby improving the patient's walking ability (42). A study by Yeo et al. (72) suggested that the combination of visual feedback training and PT improves postural stability, thereby enhancing gait kinematic variables. Research has pointed out that the prerequisite for maintaining postural stability is the normal functioning of sensory systems such as proprioception, the visual system, and the vestibular organs. A deficiency in any of these systems can lead to varying degrees of postural instability, subsequently reducing walking ability (83). Visual feedback training utilizes light to provide feedback on body movements, which can be used to compensate for the reduced position and joint sense caused by lower limb dysfunction in stroke patients. PT+ visual feedback training can enhance the input of visual and proprioceptive information during walking, thereby improving postural stability and walking ability in stroke patients.

We reviewed studies on interventions PT + closed kinematic chain exercises and PT + resistance training. The studies on PT + closed kinematic chain exercises did not report the stroke onset stage, whereas the studies on PT + resistance training reported an onset stage of 4.1 days. Therefore, we recommend using PT + resistance training for treating lower limb dysfunction in stroke patients within 5 days of onset.

However, our study has certain limitations. Due to the limited number of studies included for some rehabilitation interventions, The included literature is all from the Asian region, the age of stroke patients in the included studies ranged from 45 to 70 years, and the lack of direct comparisons between different intervention regimens, the results may be subject to some bias. The results of this study are limited to the data analysis of the included literature and may not accurately reflect the true efficacy of clinical treatments. Caution is advised when referencing these findings for recommending treatment regimens. Further validation through high-quality, large-sample, multicenter RCTs with long-term follow-up is needed. We look forward to future research focusing more on older stroke patients using PT combination regimens for the treatment of lower limb dysfunction.

## 5 Conclusion

In conclusion, PT + MRP was the optimal rehabilitation regimen for improving balance ability in stroke patients; PT + CKCE was the best for enhancing lower limb motor function; and PT + VFT was most effective for improving walking ability. Overall, PT + CKCE and PT + RT1 represented the most effective interventions for lower limb functional rehabilitation in stroke patients, while PT + RT1 is most effective within 5 days of stroke onset.

## Data Availability

The original contributions presented in the study are included in the article/Supplementary material, further inquiries can be directed to the corresponding author.
