# Supplementary material for: Optimal proprioceptive training combined with rehabilitation regimen for lower limb dysfunction in stroke patients: a systematic review and network meta-analysis
Source: Front Neurol. 2024 Dec 20;15:1503585. doi: 10.3389/fneur.2024.1503585 (PMC11695241; doi:10.3389/fneur.2024.1503585)
Supplement: Supplementary file 1 [file Data_Sheet_1.docx]

**Supplementary Table S1** Search strategy

**Pubmed**

**#1** " Proprioception "[Mesh]

**#2** Vestibular Sense OR Equilibrium Sense OR Labyrinthine Sense OR Position Sense OR Posture Sense OR deep sensitivity OR kinaesthetic discrimination OR kinaesthetic perception OR kinesio perceptual test OR kinesthetic discrimination OR kinesthetic perception OR kinetic tonic pattern OR propriocep* OR proprioceptive training OR proprioceptive balance OR proprioceptive balance board training OR sensorimotor training [Title/Abstract]

**#3** #1 OR #2

**#4** " Stroke "[Mesh]

**#5** Stroke* OR Cerebrovascular Accident* OR CVA* OR Brain Vascular Accident* OR Apoplex* OR acute cerebrovascular lesion OR acute focal cerebral vasculopathy OR brain accident OR brain attack OR brain blood flow disturbance OR brain insult* OR cerebral insult OR cerebrovascular arrest OR cerebrovascular failure OR cerebrovascular injury OR cerebrovascular insufficiency OR cerebrovascular insult OR cerebrum vascular accident OR insultus cerebralis OR ischaemic seizure OR ischemic seizure [Title/Abstract]

**#6** #4 OR #5

**#7** Random* [Title/Abstract]

**#8** #3 AND #6 AND #7

**Cochrane Library**

**#1** MeSH descriptor: [Proprioception] explode all trees

**#2** Vestibular Sense OR Equilibrium Sense OR Labyrinthine Sense OR Position Sense OR Posture Sense OR deep sensitivity OR kinaesthetic discrimination OR kinaesthetic perception OR kinesio perceptual test OR kinesthetic discrimination OR kinesthetic perception OR kinetic tonic pattern OR propriocep* OR proprioceptive training OR proprioceptive balance OR proprioceptive balance board training OR sensorimotor training :ti,ab,kw

**#3** #1 OR #2

**#4** MeSH descriptor: [Stroke] explode all trees

**#5** Stroke* OR Cerebrovascular Accident* OR CVA* OR Brain Vascular Accident* OR Apoplex* OR acute cerebrovascular lesion OR “acute focal cerebral vasculopathy OR brain accident OR brain attack OR brain blood flow disturbance OR brain insult* OR cerebral insult OR cerebrovascular arrest OR cerebrovascular failure OR cerebrovascular injury OR cerebrovascular insufficiency OR cerebrovascular insult OR cerebrum vascular accident OR insultus cerebralis OR ischaemic seizure OR ischemic seizure :ti,ab,kw

**#6** #4 OR #5

**#7** Random* :ti,ab,kw

**#10** #3 AND #6 AND #7

**Web of Science**

TS=( Proprioception OR Vestibular Sense OR Equilibrium Sense OR Labyrinthine Sense OR Position Sense OR Posture Sense OR deep sensitivity OR kinaesthetic discrimination OR kinaesthetic perception OR kinesio perceptual test OR kinesthetic discrimination OR kinesthetic perception OR kinetic tonic pattern OR propriocep* OR proprioceptive training OR proprioceptive balance OR proprioceptive balance board training OR sensorimotor training ) AND TS=( Stroke* OR Cerebrovascular Accident* OR CVA* OR Brain Vascular Accident* OR “Apoplex*” OR “acute cerebrovascular lesion” OR “acute focal cerebral vasculopathy” OR brain accident OR brain attack OR brain blood flow disturbance OR brain insult* OR cerebral insult OR cerebrovascular arrest OR cerebrovascular failure OR cerebrovascular injury OR cerebrovascular insufficiency OR cerebrovascular insult OR cerebrum vascular accident OR insultus cerebralis OR ischaemic seizure OR ischemic seizure ) AND TS=( Random*)

**Embase**

**#1** 'proprioception'/exp OR 'vestibular sense':ab,ti OR 'equilibrium sense':ab,ti OR 'labyrinthine sense':ab,ti OR 'position sense':ab,ti OR 'posture sense':ab,ti OR 'deep sensitivity':ab,ti OR 'kinaesthetic discrimination':ab,ti OR 'kinaesthetic perception':ab,ti OR 'kinesio perceptual test':ab,ti OR 'kinesthetic discrimination':ab,ti OR 'kinesthetic perception':ab,ti OR 'kinetic tonic pattern':ab,ti OR propriocep*:ab,ti OR 'proprioceptive training':ab,ti OR 'proprioceptive balance':ab,ti OR 'proprioceptive balance board training':ab,ti OR 'sensorimotor training':ab,ti

**#2** 'cerebrovascular accident'/exp OR stroke*:ab,ti OR 'cerebrovascular accident*':ab,ti OR cva*:ab,ti OR 'brain vascular accident*':ab,ti OR apoplex*:ab,ti OR 'acute cerebrovascular lesion':ab,ti OR 'acute focal cerebral vasculopathy':ab,ti OR 'brain accident':ab,ti OR 'brain attack':ab,ti OR 'brain blood flow disturbance':ab,ti OR 'brain insult*':ab,ti OR 'cerebral insult':ab,ti OR 'cerebrovascular arrest':ab,ti OR 'cerebrovascular failure':ab,ti OR 'cerebrovascular injury':ab,ti OR 'cerebrovascular insufficiency':ab,ti OR 'cerebrovascular insult':ab,ti OR 'cerebrum vascular accident':ab,ti OR 'insultus cerebralis':ab,ti OR 'ischaemic seizure':ab,ti OR 'ischemic seizure':ab,ti

**#3** random*:ab,ti

**#4** #1 AND #2 AND #3

**VIP**

（本体感觉OR本体感觉训练）AND（脑卒中OR卒中OR脑血管疾病OR中风OR脑血管中风OR脑血管意外OR偏瘫）AND（随机）

**CNKI**

（本体感觉OR本体感觉训练）AND（脑卒中OR卒中OR脑血管疾病OR中风OR脑血管中风OR脑血管意外OR偏瘫）AND（随机）

**Wanfang**

（本体感觉OR本体感觉训练）AND（脑卒中OR卒中OR脑血管疾病OR中风OR脑血管中风OR脑血管意外OR偏瘫）AND（随机）

**Sinomed**

**#1** "本体感觉"[不加权:扩展]

**#2** "卒中"[不加权:扩展]

**#3** ( "脑卒中"[常用字段:智能] OR "卒中"[常用字段:智能] OR "脑血管疾病中风"[常用字段:智能] OR "脑血管中风"[常用字段:智能] OR "脑血管意外"[常用字段:智能] OR "偏瘫"[常用字段:智能])

**#4** ( "本体感觉"[常用字段:智能] OR "本体感觉训练"[常用字段:智能])

**#5** "随机"[摘要:智能]

**#6** (#4) OR (#1)

**#7** (#3) OR (#2)

**#8** (#7) AND (#6) AND (#5)
